# Relationship between social participation and depressive symptoms in patients with multimorbidity: the chained mediating role of cognitive function and activities of daily living

**DOI:** 10.1186/s12889-024-19157-7

**Published:** 2024-07-10

**Authors:** Huaiju Ge, Shihong Dong, Wenyu Su, Weimin Guan, Qing Yu, Yan Liu, Yuantao Qi, Xialing Sun, Huiqing Zhang, Guifeng Ma

**Affiliations:** 1 School of Public Health, Shandong Second Medical University, Weifang, Shandong China; 2 Shandong Cancer Research Institute (Shandong Tumor Hospital), Jinan, Shandong China; 3 The First Affiliated Hospital of Shandong Second Medical University (Weifang People’s Hospital), Weifang, Shandong China

**Keywords:** Multimorbidity, Social participation, Depressive symptoms, Cognitive function, Activities of daily living

## Abstract

**Objective:**

The potential mechanisms linking social participation and depressive symptoms in Chinese individuals with multimorbidity are not yet fully understood. This study aims to explore how cognitive function and activities of daily living (ADLs) mediate the relationship between social participation and depressive symptoms in individuals with multimorbidity.

**Methods:**

We selected 3782 participants with multimorbidity from the 2018 China Health and Retirement Longitudinal Study. Data related to social participation, cognitive function, ADLs, and depressive symptoms were extracted. Regression and Bootstrap analyses were used to explore the sequential mediating effects of social participation, cognitive function, ADLs, and depressive symptoms.

**Results:**

(1) There was a significant correlation between social participation, cognitive function, activities of daily living, and depressive symptoms (*p* < 0.01). (2) Social participation directly affected depressive symptoms (β = -0.205, *p* < 0.05). (3) Cognitive function (β = -0.070, *p* < 0.01) and activities of daily living (β = -0.058, *p* < 0.01) played separate mediating roles in the effect of social participation on depressive symptoms. (4) Cognitive function and activities of daily living had a chain-mediated role in the relationship between social participation and depressive symptoms in patients with multimorbidity (β = -0.020, *p* < 0.01).

**Conclusion:**

A chained mediating effect was found between cognitive function, ADLs, social participation, and depressive symptoms in patients with multimorbidity. Social participation was found to improve the cognitive function of patients with multimorbidity, which in turn enhanced their daily life activities and ultimately alleviated their depressive symptoms.

## Background

According to the World Health Organization (WHO), approximately 41 million people die from chronic diseases globally each year, making it one of the leading causes of death worldwide. By 2030, chronic diseases are projected to emerge as the predominant health burden in low-income and middle-income countries. Given the interconnected risk factors and pathogenic mechanisms of numerous chronic diseases, it is not uncommon for patients to have multiple chronic conditions. Multimorbidity refers to the coexistence of two or more chronic diseases in an individual [1]. Previous studies have found that patients with multimorbidity have significantly higher mortality rates, hospitalization rates, healthcare costs, and disease burden compared to those with a single chronic disease [2–4]. Studies indicate that the prevalence of multimorbidity in individuals aged 65 and older spans from 55 to 98%, presenting a significant global health challenge [5].

Previous studies [6, 7] have shown that chronic diseases serve as risk factors for mental health problems. Individuals with multimorbidity, confronted with treatment challenges, high costs, and poor prognosis, are more susceptible to depression and anxiety compared to those with a single chronic disease [8, 9]. The WHO estimates that by 2023, depression will emerge as a leading contributor to the global disease burden [10, 11]. Depression is a severe mental disorder characterized by persistent sadness, loss of interest and pleasure, fatigue, self-deprecation, and potentially suicidal tendencies. It significantly influences an individual’s emotions, thoughts, and behaviors, disrupting normal daily life. As individuals age, symptoms of depression may worsen, potentially triggering the onset of dementia [12]. Furthermore, individuals with depression may experience insomnia, reduced cognitive abilities, and loss of appetite due to prolonged negative emotions. This makes it challenging to manage their chronic conditions, potentially exacerbating their health issues and creating a vicious cycle.

Long-term depression significantly adversely affects an individual’s work, life, and interpersonal relationships [13, 14]. Social participation is one of the effective ways to alleviate depressive emotions, and active engagement in social activities serves as a protective factor against depression [15–17]. Social participation refers to the active engagement of individuals or groups in social activities and affairs, exerting a positive influence on both individuals and society. Social participation plays a crucial role in the active aging process [13]. Active participation in social activities enables individuals to expand their social networks, acquire social support, cultivate a sense of belonging, enhance psychological resilience, boost self-esteem and confidence, increase life satisfaction, and feel valuable to society. This, in turn, alleviates depressive feelings and enhances the overall quality of life.

Cognitive ability encompasses an individual’s mental capacity, including abilities in thinking, memory, learning, understanding, reasoning, and problem-solving. Some studies have found a correlation between social participation and cognitive function [18–20]. Active engagement in social activities can enhance the effective utilization of brain network reserves, thereby compensating for cognitive function [21]. For instance, activities such as playing mahjong, chess, or cards among middle-aged and elderly individuals can enhance reasoning and judgment abilities, promote the connection between brain neurons, and facilitate the formation of new synapses [22], thus delaying the progression of cognitive decline [23, 24]. Cognitive decline may result in impaired daily functioning, diminished self-care abilities, gradual decline in memory capacity, restricted social interactions, and an increased likelihood of experiencing self-worth negation and helplessness, eventually leading to depressive symptoms [25].

Furthermore, the capacity to perform activities of daily living serves as a crucial predictor of social participation [26]. Improving an individual’s ability to perform activities of daily living can boost their social participation, and active social participation also contributes to promoting an individual’s ability to perform activities of daily living [27, 28]. Social participation can help individuals access health services and health-related information through interactive communication [29], mitigating against adverse outcomes [30]. Previous research has demonstrated that a low level of ability to perform activities of daily living constitutes a risk factor for depressive symptoms [31, 32]. Individuals with lower abilities to perform activities of daily living often require long-term care from family members or others, leading to a loss of independence and an increased likelihood of experiencing negative emotions such as low self-esteem and depression.

**Fig. 1:**
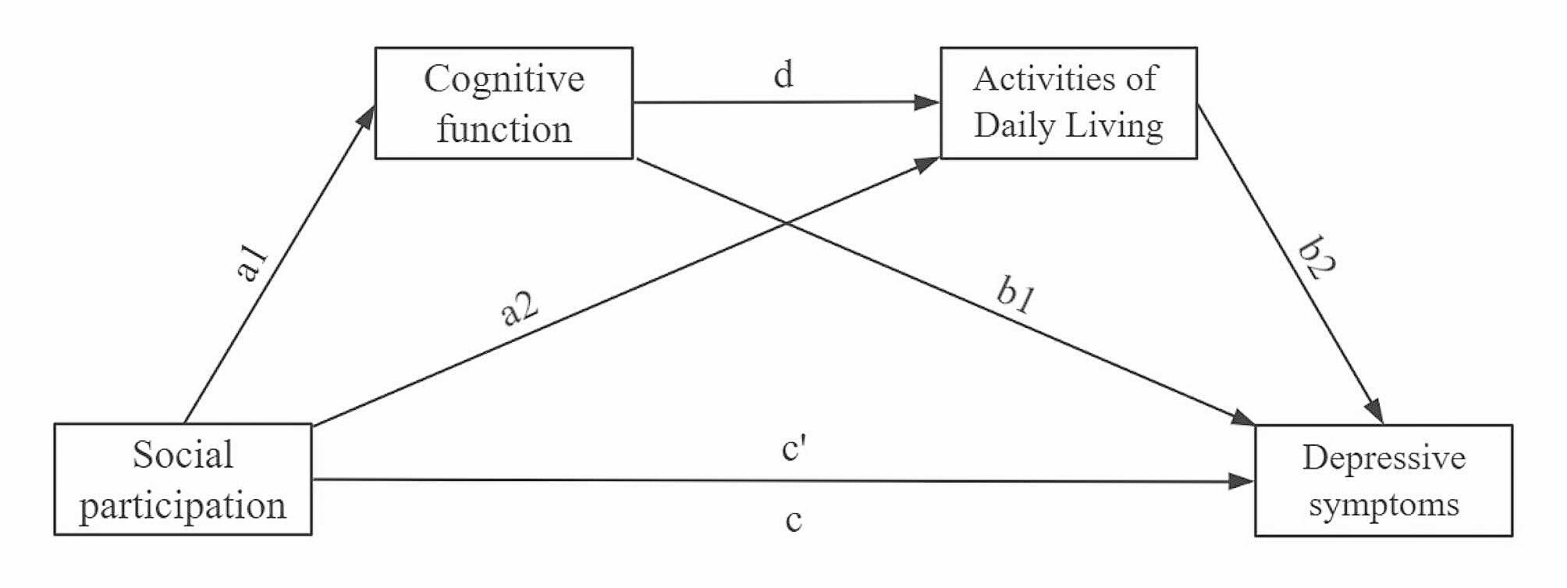
The hypothesized chain mediation effect model

Although there is existing research on the relationship between social participation and depressive symptoms in middle-aged and elderly individuals [13, 16, 33], there is relatively limited research on how social participation influences depressive symptoms in patients with multiple chronic conditions, particularly studies related to potential chain mediation effects. The current prevalence of multiple chronic conditions remains at a high level [34, 35], social participation may adversely affect depressive symptoms by affecting cognitive function and levels of activities of daily living in people with multiple chronic diseases. Therefore, this study aims to investigate the relationship between social participation and depressive symptoms in patients with multiple chronic conditions and to explore potential mechanisms from social, psychological, and physiological perspectives. This study hypothesizes that in a chain mediation model, social participation indirectly delays the development of depressive symptoms in patients with multiple chronic conditions through elevated levels of cognitive ability and ADLs. Based on previous research, we propose the following hypotheses (Fig. 1).

### Hypothesis 1:

Social participation negatively predicts depressive symptoms in patients with multiple chronic conditions (c);

### Hypothesis 2:

Cognitive function mediates the relationship between social participation and depressive symptoms (a1, b1);

### Hypothesis 3:

Activities of daily living mediate the relationship between social participation and depressive symptoms (a2, b2);

### Hypothesis 4:

Cognitive function and activities of daily living moderate the relationship between social participation and depressive symptoms through a chain mediator model (a1, d, b2).

## Materials and methods

### Sample and data collection

Data for this study was obtained from the China Health and Retirement Longitudinal Study (CHARLS). CHARLS is a long-term tracking survey project jointly initiated by the Institute of Sociology of the Chinese Academy of Social Sciences and the National School of Development at Peking University. The project commenced in 2010 and employed a multi-stage random sampling method with stratified processing of county-level units nationwide, aiming to understand the trends and changes in China’s socio-economic development through a longitudinal study of Chinese households. The survey subjects are mainland Chinese residents, and the survey covers various aspects including basic demographic information, family background, health status, economic income, education level, and labor force participation. The survey is conducted through face-to-face interviews, with a follow-up survey conducted every two years. CHARLS involves a total of 10,257 households and 17,708 individuals. The research has been approved by the Peking University Institutional Review Board (IRB 00001052–11,015), and informed consent has been obtained from each participant. It represents a high-quality micro dataset of Chinese households and individuals aged 45 and above.

The data for this study was obtained from the 4th wave of the national sample of the CHARLS in 2018. A total of 3782 subjects were ultimately included based on the following criteria: (1) Participants had multiple chronic diseases, defined as any combination of 13 specific chronic conditions, including hypertension, dyslipidemia, diabetes, cancer, chronic lung disease, liver disease, heart disease, stroke, kidney disease, gastrointestinal diseases, emotional and mental health issues, arthritis or rheumatic diseases, and asthma; (2) Subjects with memory-related diseases (such as Alzheimer’s disease, brain atrophy, Parkinson’s disease) and physical disabilities were excluded; (3) No missing data on sociodemographic characteristics, socioeconomic status, and health-related variables; (4) No missing information on the scores from the Center for Epidemiologic Studies Depression Scale, Mini-Mental State Examination, and Activities of Daily Living assessment scale.

### Measures

#### Social participation

To assess the level of social participation, we extracted information from the CHARLS database on the social activities in which the respondents participated in the past month. The selected social participation activities included: (1) visiting friends or relatives; (2) playing mahjong, chess, or cards, or attending community activities; (3) providing help to non-cohabitating relatives, friends, or neighbors; (4) dancing, exercising, or practicing qigong; (5) participating in club or organization activities; (6) engaging in volunteer or charity work; (7) caring for non-cohabitating patients or disabled individuals; (8) attending school or training courses; (9) trading stocks (funds and other financial securities); (10) using the internet; (11) other social activities. Each activity was assigned one point, resulting in a total score ranging from 0 to 11. A higher score indicated a higher level of social participation.

### Depressive symptoms

A simplified version of the Center for Epidemiologic Studies Depression Scale (CESD-10) was used to measure the depressive symptoms of patients with multiple chronic conditions, which has good reliability and validity and has been validated and applied in the Chinese elderly population [36]. CESD-10 comprises 10 items: (1) I felt bothered by things that usually don’t bother me; (2) I had trouble keeping my mind on what I was doing; (3) I felt depressed; (4) I felt that everything I did was an effort; (5) I felt hopeful about the future; (6) I felt fearful; (7) My sleep was restless; (8) I was happy; (9) I felt lonely; (10) I could not get going. Respondents were required to self-assess the frequency of their feelings and behaviors over the past week and assign scores of 0, 1, 2, or 3 based on “rarely or none of the time (< 1 day)”, “some or a little of the time (1–2 days)”, “occasionally or a moderate amount of time (3–4 days)”, and “most or all of the time (5–7 days)”. Notably, items 5 and 8 are positively framed and thus require reverse scoring. The total score ranges from 0 to 30, with higher scores indicating more severe depressive symptoms. A score of ≥ 10 is considered indicative of depressive symptoms [37]. In this study, the CESD-10 had good reliability (Cronbach’s alpha = 0.798) and validity (KMO = 0.879, *p* < 0.01).

### Cognitive function

The Mini-Mental State Examination (MMSE) was used to assess the cognitive function of patients with multiple chronic conditions, encompassing dimensions such as orientation, memory, attention, calculation, language comprehension, and expression. Orientation primarily evaluated the respondents’ temporal and spatial orientation, with a total score of 10. Memory assessment involved immediate and delayed recall of three phrases (ball, flag, and tree), with a total score of 6. Attention and calculation required participants to perform simple calculations (e.g., subtracting 7 from 100, repeated five times), with a total score of 5. Language abilities were assessed through naming, repetition, reading, three-stage command comprehension (e.g., “Please pick up the paper with your right hand, fold it in half with both hands, and place it on your left thigh”), writing, and construction, with a total score of 9. Each correct answer scored one point, resulting in a total score ranging from 0 to 30, with higher scores indicating better cognitive function. Good reliability (Cronbach’s alpha = 0.738) and validity (KMO = 0.813, *p* < 0.01) of MMSE.

### Activities of daily living

The Activities of Daily Living scale (ADLs) was used to comprehensively evaluate the respondents’ ability to perform daily living activities. The ADLs has acceptable reliability (Cronbach’s alpha = 0.794) and validity (KMO = 0.876, *p* < 0.01). The measurement of daily living activities was divided into basic activities of daily living (BADL) and instrumental activities of daily living (IADL). BADL included toileting, eating, dressing, controlling bowel and bladder, getting in and out of bed, and bathing. IADL included shopping, using the telephone, cooking, housekeeping, taking medication, and managing finances. Respondents were asked to choose “no difficulty”, “some difficulty but still able to complete”, “difficulty and need assistance”, or “unable to complete” based on their situation, with scores of 1, 2, 3, or 4 assigned, respectively. The total score ranged from 12 to 48, with lower scores indicating higher levels of daily living activity.

### Covariates

Based on the existing research [36, 38–40], we included several potential influencing factors as control variables in the relationship between social participation and depressive symptoms among patients with multiple chronic conditions. These factors encompass sociodemographic characteristics, socioeconomic status, and health-related variables. Sociodemographic characteristics consist of age (years), gender (1 = male, 2 = female), and marital status (1 = married/cohabiting, 2 = married/living separately, 3 = divorced, 4 = widowed, 5 = unmarried). Socioeconomic status includes educational level (1 = no education, 2 = primary school or below, 3 = middle school, 4 = high school and above), residential area (0 = rural, 1 = urban), employment status (0 = unemployed, 1 = employed), and geographical distribution (1 = eastern region, 2 = central region, 3 = western region). Geographical distribution is based on the theory of regional economic development in China, dividing provinces into eastern, central, and western regions. Health-related variables comprise self-rated health, sleep duration (hours), life satisfaction, and physical exercise (0 = no, 1 = yes). Physical exercise refers to a daily exercise duration of ≥ 30 min.

### Statistical analysis

The data for this study were analyzed using SPSS 25.0 for statistical analysis. Initially, a descriptive statistical analysis of the variables was conducted, using (M ± SD) to describe the metric data, and using percentages or proportions to describe the count data. Differences in characteristics between different depressive symptom subgroups were compared using t-tests or chi-square tests [41]. Subsequently, the correlation between the research variables was examined. Finally, a chained mediation analysis was performed using Model 6 in the PROCESS 4.0 macro. After controlling for covariates, the level of social participation was designated as the independent variable (X), depressive symptoms as the outcome variable (Y), and cognitive function (M1) and activities of daily living (M2) as the mediating variables. The mediation effects were tested and confidence intervals were estimated using the Bootstrap method with 5000 resamples, and the significance of direct, indirect, and total effects was determined based on the confidence intervals (whether they included 0 or not). We considered the differences to be statistically significant at *p* < 0.05.

## Result

### Descriptive statistical analysis and correlation analysis

Using a CES-D score of ≥ 10 as the threshold, the detection rate of depressive symptoms among Chinese patients with multiple chronic conditions was 47.28% (1788 individuals). The basic information of 3782 patients with multiple chronic conditions in this study is presented in Table 1.

**Table 1:** Descriptive statistical analysis(*n* = 3782)

Variables	*n*	%	Depressive Symptoms		M ± SD	Rang
			M ± SD	*p*		
Age(years)					68.43 ± 6.08	60 ~ 92
Sleep duration(hours)					5.86 ± 2.07	0 ~ 15
Gender				< 0.01		
Male	1704	45.06	9.02 ± 6.35			
Female	2078	54.94	11.02 ± 7.06			
Educational level				< 0.01		
Uneducated	976	25.81	12.04 ± 7.17			
Primary and below	1809	47.83	10.32 ± 6.81			
Junior high school	605	16.00	8.42 ± 5.78			
High school and above	392	10.36	7.04 ± 5.64			
Marital status				< 0.01		
Married cohabiting	2955	78.13	9.72 ± 6.66			
Married separated	119	3.15	10.55 ± 6.84			
Divorce	45	1.19	12.11 ± 7.67			
Widowhood	647	17.11	11.72 ± 7.18			
Unmarried	16	0.42	10.56 ± 7.45			
Residence				< 0.01		
Rural	2172	57.43	11.31 ± 6.86			
Urban	1610	42.57	8.51 ± 6.42			
Physical exercise				< 0.01		
Yes	1976	52.25	9.44 ± 6.55			
No	1806	47.75	10.86 ± 7.02			
Geographic distribution				< 0.01		
Eastern Region	1187	31.39	8.88 ± 6.51			
Central Region	1324	35.00	10.27 ± 6.79			
Western Region	1271	33.61	11.12 ± 6.95			
Job status				< 0.01		
Employed	1823	48.20	10.44 ± 6.77			
Unemployed	1959	51.80	9.82 ± 6.85			
Self-assessed health				< 0.01		
Very Good	173	4.57	6.21 ± 5.73			
Good	222	5.87	6.71 ± 5.91			
Fair	1829	48.36	8.77 ± 6.09			
Poor	1186	31.36	12.04 ± 6.76			
Very Poor	372	9.84	14.47 ± 7.40			
Satisfaction with life				< 0.01		
Completely Satisfied	162	4.28	7.44 ± 6.05			
Very Satisfied	1070	28.29	7.60 ± 6.03			
Somewhat Satisfied	2091	55.29	10.09 ± 6.22			
Not Very Satisfied	339	8.96	16.07 ± 6.56			
Not at All Satisfied	120	3.17	19.93 ± 5.86			
Depressive Symptoms					10.12 ± 6.82	0 ~ 30
Cognitive Function					21.16 ± 5.29	1 ~ 30
Social Participation					0.82 ± 1.05	0 ~ 11
Activities of Daily Living					14.49 ± 4.24	12 ~ 48

Social participation was found to have a negative correlation with depressive symptoms among patients with multiple chronic conditions, while cognitive function exhibited a negative correlation with depressive symptoms. Conversely, daily living activities showed a positive correlation with depressive symptoms. Furthermore, social participation was positively correlated with cognitive function but negatively correlated with daily living activities. Finally, cognitive function was negatively correlated with daily living activities. The correlations of the study variables are shown in Table 2.

**Table 2:** Correlation analysis of variables

Variables	1	2	3	4	5	6	7	8	9	10	11	12	13	14	15
1. Social participation	—														
2. Depressive symptoms	-0.139^**^	—													
3. Cognitive function	0.243^**^	-0.276^**^	—												
4. Activities of Daily Living	-0.164^**^	0.301^**^	-0.283^**^	—											
5. Age	-0.088^**^	-0.040^*^	-0.106^**^	0.167^**^	—										
6. Gender	0.036^*^	0.146^**^	-0.255^**^	0.066^**^	-0.058^**^	—									
7. Educational level	0.230^**^	-0.227^**^	0.560^**^	-0.184^**^	-0.063^**^	-0.292^**^	—								
8. Marital status	-0.015	0.112^**^	-0.158^**^	0.088^**^	0.233^**^	0.167^**^	-0.098^**^	—							
9. Residence	0.159^**^	-0.203^**^	0.281^**^	-0.123^**^	0.064^**^	0.031	0.291^**^	-0.006	—						
10. Self-assessed health	-0.088^**^	0.327^**^	-0.056^**^	0.281^**^	-0.018	-0.025	-0.066^**^	0.018	-0.118^**^	—					
11. Sleep duration	0.024	-0.273^**^	0.087^**^	-0.104^**^	-0.001	-0.158^**^	0.078^**^	-0.077^**^	0.019	-0.133^**^	—				
12. Physical exercise	0.128^**^	-0.104^**^	0.135^**^	-0.143^**^	-0.027	0.019	0.094^**^	-0.007	0.134^**^	-0.033^*^	-0.008	—			
13. Geographic distribution	-0.052^**^	0.132^**^	-0.116^**^	0.035^*^	0.002	-0.040^*^	-0.076^**^	0.016	-0.058^**^	0.058^**^	-0.080^**^	-0.003	—		
14. Job status	-0.063^**^	0.046^*^	-0.080^**^	-0.151^**^	-0.257^**^	-0.102^**^	-0.131^**^	-0.139^**^	-0.323^**^	-0.050^**^	0.007	-0.061^**^	0.065^**^	—	
15. Satisfaction with life	-0.037^*^	0.389^**^	-0.042^**^	0.121^**^	-0.099^**^	0.011	0.013	0.033^*^	-0.054^**^	0.221^**^	-0.129^**^	-0.046^**^	0.022	0.022	—

Note*p < 0.05, **p **< 0.01**

### Analysis of chain mediation effects

Based on the results of descriptive and correlational analyses, the present study used a chain mediated effects model controlling for age, gender, education level, marital status, residence, self-assessed health, sleep duration, physical exercise, geographic distribution, job status, and satisfaction with life to further examine the mediating roles of cognitive function and activities of daily living in the association between social participation and depressive symptoms among patients with multiple chronic diseases. As shown in Table 3, social participation level had a negative predictive effect on depressive symptoms among patients with multiple chronic conditions (*β* = −0.205, *p* < 0.05), which supported Hypothesis 1. After incorporating cognitive function and daily living activities into the structural equation model, social participation was found to positively predict cognitive function (*β* = 0.537, *p* < 0.01) and negatively predict daily living activities (*β* = −0.258, *p* < 0.01). High levels of cognitive function were significantly associated with poor daily living activities (*β* = −0.166, *p* < 0.01) and fewer depressive symptoms (*β* = −0.130, *p* < 0.01), while daily living activities had a positive predictive effect on depressive symptoms (*β* = 0.225, *p* < 0.01).

**Table 3:** Regression analysis of social participation, cognitive function, and ADLs on depressive symptoms

Independent Variable	Cognitive Function(M1)			ADLs(M2)			Depressive Symptom(Y)		
		β	t		β	t		β	t
Social participation(X)	a1	0.537^***^	7.914	a2	-0.258^***^	-4.188	c’	-0.205^*^	-2.273
Cognitive function(M1)				d	-0.166^***^	-11.330	b1	-0.130^***^	-5.968
ADLs(M2)							b2	0.225^***^	9.469
Age		-0.062^***^	-5.091		0.076^***^	6.931		-0.054^**^	-3.333
Gender		-1.370^***^	-9.100		0.008	0.055		0.905^***^	4.521
Educational level		2.551^***^	29.690		-0.110	-1.284		-0.466^***^	-3.712
Marital status		-0.328^***^	-5.306		-0.025	-0.454		0.316^***^	3.875
Residence		1.388^***^	9.034		-0.552^***^	-3.939		0.635^***^	5.632
Self-assessed health		0.056	0.705		1.061^***^	14.947		1.321^***^	12.388
Sleep duration		0.035	1.025		-0.089^**^	-2.915		-0.531^***^	-11.870
Physical exercise		0.654^***^	4.720		-0.791^***^	-6.313		-0.441^*^	-2.401
Geographic distribution		-0.470^***^	-5.509		-0.030	-0.392		0.635^***^	5.632
Job status		-0.058	-0.380		-1.387^***^	-10.122		0.298	1.469
Satisfaction with life		-0.242^**^	-2.711		0.330^***^	4.086		2.531^***^	21.444
Fit index									
*R*	0.615^***^			0.461^***^			0.593^***^		
*R* ^*2*^	0.378			0.213			0.352		
*F*	190.689			78.270			146.000		

Note*p < 0.05, **p < 0.01, ***p **< 0.001**

**Fig. 2:**
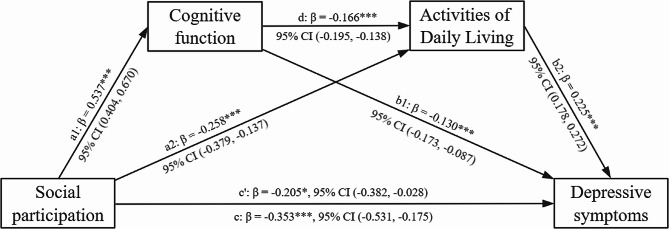
A model of the chain-mediated effects of cognitive function and activities of daily living between social participation and depressive symptoms

Using Model 6 of the SPSS PROCESS macro with covariates, the mediating effects of cognitive function and daily living activities on the relationship between social participation and depressive symptoms were tested. As shown in Table 4, the total effect was significant (*Effect* = − 0.353, 95% CI (− 0.531, − 0.175)), as were the direct effect (*Effect* = − 0.205, 95% CI (− 0.382, − 0.028)) and the indirect effect (*Effect* = − 0.148, 95% CI (− 0.192, − 0.110)). Cognitive function had a significant mediating effect on the relationship between social participation and depressive symptoms (*Effect* = − 0.070, 95% CI (− 0.101, − 0.043)), supporting Hypothesis 2. Daily living activities also had a significant mediating effect on this relationship (*Effect* = − 0.058, 95% CI (− 0.085, − 0.036)), supporting Hypothesis 3. Moreover, the chained mediation effect of cognitive function and daily living activities on the relationship between social participation and depressive symptoms was significant (*Effect* = − 0.020, 95% CI (− 0.029, − 0.013)), supporting Hypothesis 4. The chain mediating effect model of cognitive function and activities of daily living between social participation and depressive symptoms is shown in Fig. 2.

**Table 4:** Analysis of Chain mediating effects

Path	Effect	Boot SE	95% CI		Proportion(%)
			Lower	Upper	
Total effect	-0.353	0.091	-0.531	-0.175	
Direct effect of social participation	-0.205	0.090	-0.382	-0.028	58.07
Indirect effect	-0.148	0.021	-0.192	-0.110	41.93
Social participation→MMSE→CESD	-0.070	0.015	-0.101	-0.043	19.83
Social participation→ADLs→CESD	-0.058	0.013	-0.085	-0.036	16.43
Social participation→MMSE→ADLs→CESD	-0.020	0.004	-0.029	-0.013	5.67

## Discussion

The results of this study showed a negative correlation between social participation and depressive symptoms, with social participation having a negative predictive effect on depressive symptoms, which is consistent with previous research [38, 42], indicating that higher levels of social participation are associated with milder depressive symptoms. Participating in social activities can help regulate personal emotions, obtain social support, meet spiritual needs, prevent, and alleviate negative emotions such as depression, and improve mental health.

This study found that social participation among older Chinese individuals remains low [43, 44], possibly due to entrenched traditional Chinese beliefs. In China, the family holds a central place in people’s lives, deeply ingrained in their beliefs, requiring considerable time and effort to sustain. Some studies [45, 46] have indicated that residing in an extended family serves as a protective factor against depressive symptoms in Europeans. However, other studies [47, 48] suggest that living in an extended family may not positively impact mental health; rather, Chinese older adults residing with their adult children are more susceptible to depressive symptoms. This phenomenon could be attributed to cultural disparities between the East and the West. Upon reaching retirement age, Chinese older adults often prioritize assisting their family members, particularly their children, with daily responsibilities, such as household chores, childcare, and financial support [49], leaving little personal time for their own pursuits. This lack of personal time, coupled with limited social interaction and spiritual support, can potentially result in feelings of loneliness, helplessness, and diminished self-worth [47], consequently heightening the risk of depression.

Furthermore, multiple chronic diseases may accelerate this process. Chronic diseases such as diabetes arthritis and heart disease can lead to restricted social participation in patients, and as the number of chronic diseases increases, the risk of impaired social participation abilities in patients increases, leading to the emergence of negative emotions such as depression and anxiety [50]. A survey of 16,032 community-dwelling elderly population aged 65 years or older in Korea also showed that chronic diseases can limit the level of daily activities and increase psychological stress in the elderly population [51]. Compared to a single chronic disease, the pathogenesis of multiple chronic diseases is more complex and often accompanied by abnormal activation of the immune system and inflammation [52–54]. For example, hypertension combined with arthritis, type II diabetes combined with arthritis, etc., can cause long-term physical discomfort and even loss of labor capacity in patients, affecting their social participation and economic status, severely impacting their quality of life, and requiring long-term treatment and medication management, which brings a heavy economic burden to individuals and families, leading to the occurrence of depression. The risk of depression in patients with multiple chronic diseases is twice that of patients with a single chronic disease and three times that of individuals without chronic diseases [8].

This study found that there is a chain-mediated effect between cognitive function, daily living activities, social participation, and depressive symptoms in patients with multiple chronic diseases. Firstly, cognitive function mediates the relationship between social participation and depressive symptoms. Active social participation can help develop a social support network, providing patients with increased social support [19], enhancing psychological stimulation, leading to improved synaptic density and neural growth in the nervous system [55, 56], and regulating the immune and neuroendocrine systems, thereby increasing disease resistance, and reducing the risk of cognitive decline-related chronic diseases [57, 58]. Additionally, social participation can assist patients in stress reduction. Relevant research indicates [59] that stress is a risk factor for neurodegeneration, potentially leading to hippocampal atrophy or shrinkage, affecting cognitive functions such as memory and emotional regulation [60]. Cognitive decline may lead to the occurrence of neural inflammation and oxidative stress, resulting in damage to brain neurons or synapses, leading to slowed thinking, decreased ability for self-care, and the emergence of negative emotions such as anxiety and depression. Therefore, social participation may alleviate depressive symptoms by enhancing cognitive function.

Furthermore, daily living activities mediate the relationship between social participation and depressive symptoms. ADLs serve as an indicator of an individual’s functional status, and impairment in these activities can lead to a decline in the individual’s quality of life [61], affecting their mental health and potentially leading to the development of depressive symptoms. Michael et al. found that ADLs impairment is associated with lower quality of life and lack of social engagement, influencing the development of depressive symptoms [62]. Social participation acts as a protective factor for daily living activities and can reduce the risk of functional disabilities [63]. Engaging in social activities can enhance an individual’s social skills, and adaptability to life, increase physical activity, promote sleep quality, and improve daily living activities. Additionally, chronic diseases are also a significant influencing factor in daily living activities. For instance, stroke, as a disease with high mortality, disability, and recurrence rates, can cause severe motor impairments due to the neurological damage it inflicts, leading to ADLs impairment in patients [64]. Stress and anxiety are risk factors for chronic diseases [65], and active participation in social activities can help patients reduce psychological stress and improve their daily living activities. Impairment in ADLs may lead to reduced social interactions, increasing social isolation, and feelings of loneliness. Loneliness and social isolation are common risk factors for depressive moods. Therefore, social participation may alleviate depressive symptoms by enhancing daily living activities.

Finally, social participation can enhance cognitive function, and good cognition can improve daily living activities, thereby alleviating depressive symptoms in patients with multiple chronic diseases. The decline in cognitive function is a predictive factor for the decline in daily living activities [66, 67], which validates the results of our study. Active engagement in social activities has a significant impact on overall cognitive function and daily living activities in adults [66]. Additionally, multiple chronic diseases, such as hypertension combined with cardiovascular diseases, may induce vascular changes that impact cerebral blood flow, causing ischemic damage in the white matter regions [68, 69]. This can result in a decline in cognitive function among patients [70], leading to memory loss and decreased decision-making ability [71], ultimately affecting daily living activities. Good cognitive function enables individuals to better plan and organize daily life, handle various problems, improve social skills, and enhance life independence, and individual quality of life [72], thereby promoting mental health [73]. Therefore, enhancing cognitive function can help delay the decline in daily living activities in patients with multiple chronic diseases. In summary, cognitive function and daily living activities play an independent mediating role in the social participation and depressive symptoms of patients with multiple chronic diseases, and there is also a chain mediating effect.

### Implications

In conclusion, the following recommendations are proposed to improve the mental health status of patients with multimorbidity. Firstly, considering the current low level of social participation and limited social awareness in China, it is recommended that the government implement corresponding incentive policies and regularly organize volunteer services, health activities, cultural education, and other social welfare programs. This is aimed at promoting the active participation of middle-aged and elderly individuals, providing social support, establishing social networks, enhancing the sense of social participation for patients with multimorbidity, and alleviating negative emotions, such as loneliness and depression [74]. Secondly, a life-course perspective should be considered to improve or delay cognitive decline. This involves personalized cognitive training for memory, attention, executive function, and decision-making [72], enhancing cognitive reserve [75], and implementing dietary modifications [76]. Regular cognitive function assessments and screenings should be carried out in middle-aged and elderly individuals to detect, intervene, and treat early. Encouraging ongoing learning in the middle-aged and elderly can enhance cognitive reserve, promote neural networks development, connections, and maintenance, thereby protecting cognitive function [75]. Additionally, promoting a Mediterranean dietary regimen, which emphasizes higher consumption of fruits and vegetables and lower intake of high-sugar and high-fat foods, can help postpone cognitive decline in patients with multiple chronic conditions [77] and decrease the risk of chronic diseases like diabetes, cardiovascular disease, and chronic lung disease [78, 79]. Thirdly, targeted daily life skills training for patients with multimorbidity should be conducted, along with the development of corresponding physical therapy and rehabilitation training programs [80]. Supportive policies should be introduced to help patients renovate their home environment, increase assistive facilities, and provide personalized psychological counseling and therapy services to understand the specific needs of patients, thereby assisting them in managing mental health issues.

### Limitation

The limitations of this study should be acknowledged. First, this study is based on cross-sectional data from the 2018 CHARLS, and it is not yet possible to determine the causal relationship between the variables, and the conclusions need to be verified by further longitudinal analyses. Future studies may consider analyzing the development and changes of depressive symptoms in older adults at different stages of their lives using longitudinal data from a life course perspective. Second, restricted by the research content of CHARLS, we can only extract a limited number of types of social participation, and lack relevant information such as the length of activity, which may have an impact on the mental health status of the patients. Third, there is no detailed discussion of the relationship between different combinations of multimorbidity and depressive symptoms. Finally, some of the data such as depression, chronic diseases, and other conditions were obtained using self-reported information from the respondents, which may have problems such as recall bias and reporting bias.

## Conclusion

The study findings indicate that social participation can alleviate depressive symptoms in patients with multimorbidity. Additionally, cognitive function and daily life activities were found to act as a chained mediating mechanism between social participation and depressive symptoms in patients with multimorbidity. Specifically, social participation was observed to improve the cognitive function of patients with multimorbidity, subsequently enhancing their daily life activities, and ultimately alleviating their depressive symptoms. Currently, social participation in China remains at a relatively low level, and the government should introduce corresponding policies to enhance social participation, which holds significant implications for the active and healthy aging of patients with multimorbidity.

## Data Availability

This study analyzes publicly available datasets from the China Health and Retirement Longitudinal Study. These data can be found here: https://charls.charlsdata.com/pages/data/111/zh-cn.html.
